# Passive Immunity to SARS-CoV-2 at Birth Induced by Vaccination in the First Trimester of Pregnancy

**DOI:** 10.3390/ijerph182312789

**Published:** 2021-12-03

**Authors:** Ilaria Cicalini, Claudia Rossi, Luca Natale, Maria Concetta Cufaro, Giulia Catitti, Simone Vespa, Domenico De Bellis, Giulia Iannetti, Paola Lanuti, Ines Bucci, Liborio Stuppia, Vincenzo De Laurenzi, Damiana Pieragostino

**Affiliations:** 1 Center for Advanced Studies and Technology (CAST), “G. d’Annunzio” University of Chieti-Pescara, 66100 Chieti, Italy; claudia.rossi@unich.it (C.R.); lucanatale1989@gmail.com (L.N.); maria.cufaro@unich.it (M.C.C.); catittig@gmail.com (G.C.); sv85@libero.it (S.V.); domenico.debellis@studenti.unich.it (D.D.B.); giuliaiannetti686@gmail.com (G.I.); paola.lanuti@unich.it (P.L.); ines.bucci@unich.it (I.B.); stuppia@unich.it (L.S.); delaurenzi@unich.it (V.D.L.); damiana.pieragostino@unich.it (D.P.); 2 Department of Innovative Technologies in Medicine and Dentistry, “G. d’Annunzio” University of Chieti-Pescara, 66100 Chieti, Italy; 3 Department of Psychological, Health and Territory Sciences, School of Medicine and Health Sciences, “G. d’Annunzio” University of Chieti-Pescara, 66100 Chieti, Italy; 4 Department of Pharmacy, “G. d’Annunzio” University of Chieti-Pescara, 66100 Chieti, Italy; 5 Department of Medicine and Aging Science, “G. d’Annunzio” University of Chieti-Pescara, 66100 Chieti, Italy

**Keywords:** SARS-CoV-2, vaccination in pregnancy, newborn SARS-CoV-2 immunity, spike-specific T cells

## Abstract

As is well known, the COVID-19 infection is affecting the whole world, causing a serious health, social and economic crisis. The viral infection can cause a mild or severe illness, depending on how effectively the virus is countered by the immune system. In this context, the position of pregnant women remains rather unknown. The case described here reports the immune response in a woman in good health and in her newborn son, having undergone complete vaccination during the first trimester of her pregnancy. We performed a serological assay, measuring IgG antibodies to SARS-CoV-2, by a fully automated solid phase DELFIA (time-resolved fluorescence) immunoassay in a few drops of blood, collected by a finger-prick and spotted on filter paper. The dried blood spot (DBS) sample we used is the same type of sample routinely used in a newborn screening program test. Such a simple and minimally invasive approach allowed us to monitor both the mother and the newborn soon after birth for their anti-SARS-CoV-2 IgG levels. The serological test on the DBS carried out on both mother and newborn revealed the presence of anti-SARS-CoV-2 IgG antibodies up to 7 months after vaccination in the mother, and already at 48 h of life in the newborn.

## 1. Introduction

In December 2019, uncommon cases of pneumonia of unknown etiology were reported in Wuhan (China), and, within a few months, the coronavirus disease 2019 (COVID-19), caused by acute respiratory syndrome coronavirus 2 (SARS-CoV-2), spread rapidly all over the world; in just a few months, it was defined as a pandemic [1,2]. As is known, the management of any threat of communicable diseases is measured, above all, by the ability to treat and monitor vulnerable populations, and pregnant women fall into this category as they can be more affected by respiratory diseases. Indeed, the coronavirus outbreaks of SARS-CoV and coronavirus of the Middle East respiratory syndrome (MERS-CoV) in the past two decades have affected pregnant women quite severely [3,4,5]. Vaccination is the safest and most effective way to prevent COVID-19 and its consequences [6]. The implementation of vaccination programs made it essential to carry out serological investigations for immunoglobulin G (IgG) antibodies against SARS-CoV-2 on a large scale to evaluate the expression, duration, and percentage of positive responses of the antibody. Considering that serum or plasma present a laborious sample typology when applied on a large scale, albeit the most used, capillary blood collected as dry blood spot (DBS) samples would instead present a valid alternative, especially considering population screening [7,8].

When we consider the positivity to SARS-CoV-2 of pregnant women, a strong interest is directed to the newborns. As described by Naseh and Ashrafzadeh, vertical transmission of viral particles from the mother to the infant is possible. In fact, they described the case of a newborn who tested positive for COVID-19 at 24 h of life, born to an unsuspecting and asymptomatic infected mother [9]. It is well known that antibodies can be transferred from the mother to the fetus, thus protecting newborns in the first months of their life. It is of great interest to study the maternal immune response to SARS-CoV-2 infection or to vaccination during pregnancy, as well as the extent to which maternal antibodies cross the placenta, to predict the degree of passive protection acquired by the newborn.

Toner et al. observed a lack of immunity and COVID-19 antibodies both in the fetus and after birth in a newborn whose mother was positive for COVID-19 during pregnancy, at the 27th week of gestation [10].

Instead, recent studies have shown that the anti-SARS-CoV-2 vaccine induces a strong maternal humoral IgG response which, through the placental barrier, is effectively transferred to the fetus. Indeed, anti-RBD antibodies were measured in the child’s cord blood after the maternal vaccination [11,12].

Here, we describe the case of a woman who received both doses of the BNT162b2 vaccine before knowing herself to be pregnant. Since our Laboratory Center is a COVID Center and the Neonatal Screening Center for Abruzzo Region (Italy), we had the opportunity to monitor the woman and her newborn. We performed a serological test to measure IgG antibodies against SARS-CoV-2 on DBS samples, the same one routinely used in neonatal screening tests; not only the mother vaccinated with BNT162b2, but also her infant were found to be positive for anti-S1 spike IgG at the serological test.

## 2. Case Presentation

### 2.1. Sample Collection

DBS samples from the mother were collected by the Center for Studies and Advanced Technologies (CAST), “G. d’Annunzio” of Chieti-Pescara Italy, where the “Neonatal Screening Laboratory” for the Abruzzo Region is located. DBS samples from the newborn were collected by the hospital of Chieti SS. Annunziata, Department of Neonatology, in accordance with the guidelines for the newborn screening program for congenital endocrine and metabolic diseases routinely performed in Italy. The first sample was collected at 48 h of life. A second sample was collected at 14 days of life since the mother was affected by thyroid disease. This collection was carried out in accordance with the protocol of the regional screening center for metabolic diseases of the Abruzzo region (Italy), for which newborns, presenting a condition of prematurity at birth (considering weight and/or weeks of gestation) or whose mothers have thyroid disease, undergo repeat sampling and screening tests for early detection of congenital hypothyroidism after 14 days of life. Samples were collected following the approval of the Ethics Committee No. 19 of 9 September 2021. The study was conducted in accordance with the guidelines of the Declaration of Helsinki and informed consent was obtained from the mother to perform the serological test on the newborn sample.

### 2.2. Mother Immunological Structure after Vaccination

The case described here reports the effect on the immune system of a complete vaccination schedule in a healthy 37-year-old woman and her newborn son.

Specifically, the woman received two doses of the BNT162b2 vaccine during the first trimester of pregnancy; in particular, the first dose was administered at 9 weeks of gestation, while the second dose was administered at 12 weeks of gestation.

The woman voluntarily underwent monitoring of the antibody titer on DBS samples before and after vaccination, since she was enrolled in one of our studies of the response to different vaccines. As previously described [8], this serological test measures IgG antibodies against SARS-CoV-2 by a fully automated solid-phase DELFIA (time-resolved fluorescence) immunoassay in a few drops of blood collected by a finger prick and spotted on filter paper through the use of a GSP^®^/DELFIA^®^ Anti-SARS-CoV-2 IgG kit time-resolved fluoro-immunoassay on a GSP instrument (PerkinElmer^®^, Turku, Finland). The serological test performed on a DBS appears to be reproducible, as reported in Table S1 in the Supplementary Materials. Intra- and inter-assay CV and standard deviation from the calculated values within the range of the 0.48–35 ratio are ≤6.15% and ≤14.90%, respectively.

The serological test by DBS for the detection of anti-IgG antibodies was performed on the woman before vaccination and 25 days and 7 months after the administration of the second dose of the vaccine. We measured an IgG value, expressed as the fluorescence ratio of the sample with respect to the calibrator, setting a positivity cut-off equal to 1.2. The positivity cut-off value was set using a series of samples made up of 1269 subjects, already described and published in our previous work [8].

**Figure 1:**
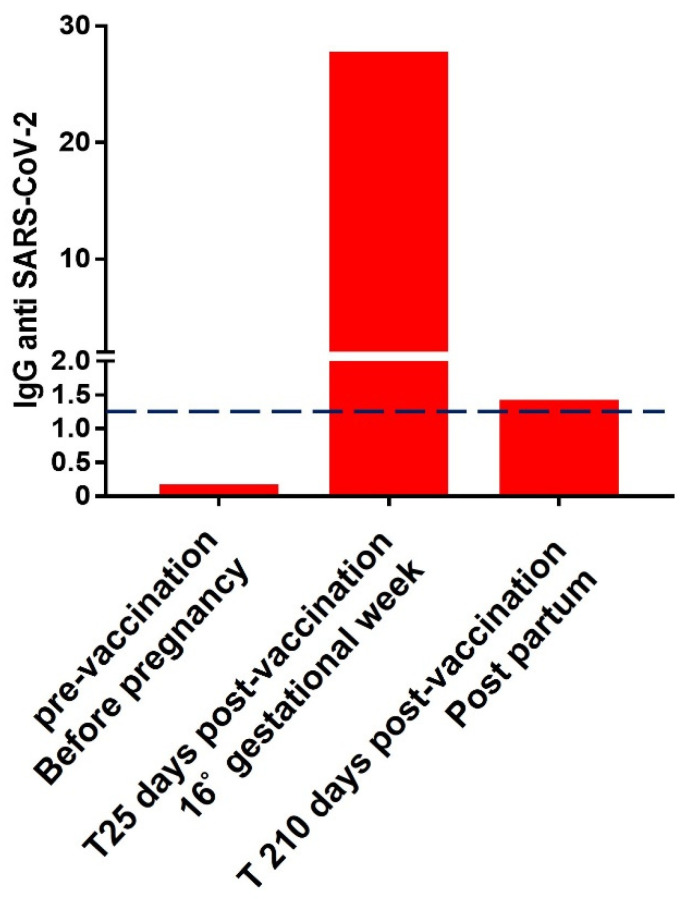
Histograms show IgG antibodies against SARS-CoV-2 levels measured on the woman’s DBS sample at 3 time points pre-vaccine administration/before pregnancy, 25 days after the second dose of vaccine administration at the sixteenth gestational week, and after 210 days (7 months) of the vaccine administration, post-partum. The positivity limit is set to 1.2, as shown by the dashed red line.

We report in Figure 1 antibody levels measured at three time points on DBS samples from the woman before and after vaccine administration.

As shown in Figure 1, the woman was antibody-naïve to SARS-CoV-2 prior to vaccination and before pregnancy with a negative antibody titer of 0.13 (cut-off 1.2). As shown in Figure 1, 25 days after completing the vaccination cycle the woman reached a full positivity, resulting in an antibody titer equal to 27.55. After 210 days, the antibody levels were, although still slightly positive, drastically decreased to a value of 1.39.

By evaluating the antibody trend of subjects undergoing complete administration of the BNT162b2 vaccine acquired in our laboratories before, then 25 days and 210 days after the complete administration of the vaccination, we observe three ranges of anti-SARS-CoV-2 IgG levels: from 0.12 to 0.26 before the vaccination, from 26.15 to 86.47 considering the second time point and from 1.43 to 7.22 for the last time point, as shown in Figure S1. These ranges were obtained by calculating the fifth and ninety-fifth percentiles in the observed population. These data show that the woman’s antibody titer was within the range observed in a larger population undergoing full vaccination and without a COVID-19 infection, and slightly below the population mean.

**Figure 2:**
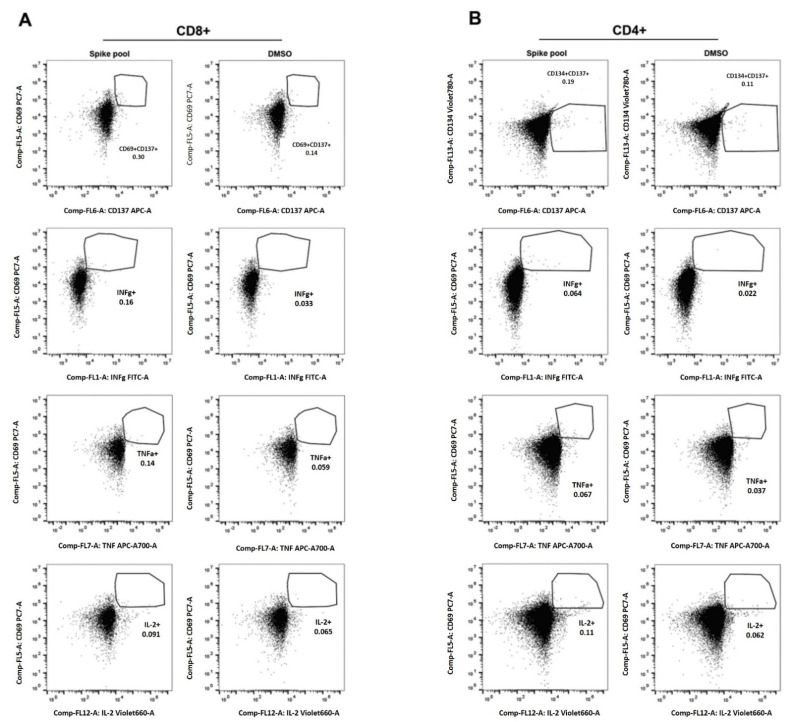
(Panel **A**) gating of CD8 + cells analyzed for CD69 and CD137 expression as well as for the production of cytokines INFg, TNFa and IL-2 in women, stimulated sample (Spike pool) vs. negative control (DMSO). (Panel **B**) gating of CD4 + cells analyzed for CD134 and CD137 as well as for the production of cytokines INFg, TNFa and IL-2 in women (Spike pool) vs. negative control (DMSO). Collection of the woman’s sample and gating analysis was performed 7 months after complete vaccination administration—post-partum.

To verify the complete immunological assessment close to the partum simultaneously with the monitoring of the anti-SARS-CoV-2 IgG levels, the woman underwent venous sampling to verify the presence and abundance of memory T cells. In order to identify spike protein (S)-specific T cells, we used the same gating strategy already described [8,13], excluding dead cells by using a cell viability dye. In particular, direct ex vivo CD4+ and CD8+ T cell responses were evaluated, analyzing key antiviral cytokine production as well as specific activation-induced markers in S-specific reactive T cells as a read-out. As reported in Figure 2, activation immune markers and cytokine production (INFg, TNFa, and IL-2) were found in CD8+ and CD4+ T cell compartments in the woman after 7 months from vaccine administration.

### 2.3. Immunological Status of the Newborn

The baby was born through a caesarean section, at 37 weeks of gestation; the birth weight was 3.350 kg, and the neonatal parameters were unremarkable. The baby boy was born approximately 25 weeks after the mother received the second vaccination dose. As the Neonatal Screening Center for the Abruzzo Region, and being at the same time a COVID Center, we had the opportunity to monitor both the woman and the newborn after childbirth. It is very important to point out that the DBS sample that we used for the neonatal serological test is the same used for the metabolic neonatal screening test. 

**Figure 3:**
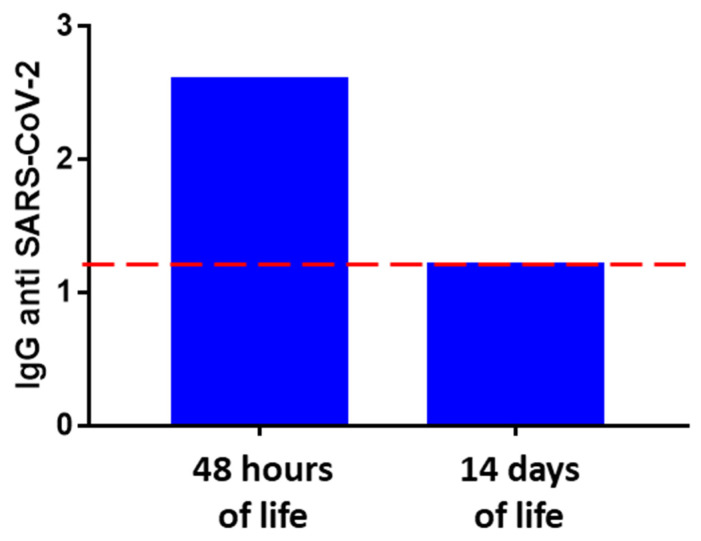
Histograms show the IgG antibodies against SARS-CoV-2 levels measured on the newborn DBS sample at 2 time points: at 48 h of life, and 14 days of life. The positivity limit is set to 1.2, as shown by the dashed red line.

As reported in the histogram in Figure 3, the serological test carried out on the DBS sample of the newborn collected at 48 h of life resulted positive, measuring an IgG value equal to 2.57 (cut-off of 1.2). The serological test repeated after 14 days of life showed an expected decrease in antibody levels, as shown in Figure 3. These results were confirmed by analyzing a second newborn whose mother underwent complete vaccination in the first trimester of pregnancy, showing the same trend, as shown in Figure S2.

## 3. Discussion

The case presented concerns a woman who received two doses of the BNT162b2 vaccine during the first trimester of a pregnancy of which she was not aware. 

As is well known, COVID-19 infection can cause mild or severe illness depending on how effectively the virus is countered by the immune system. In this context, the position of pregnant women remains rather unknown and widely discussed. Wastnedge et al. have, in fact, recently investigated this complex topic by summarizing some causes implicated in changing the immune system during pregnancy [1]. The immune system during pregnancy undergoes a series of adaptations designed to allow the growth of the fetus, and these changes are, above all, reflected on the immunological structure. In fact, it is believed that the altered immune response to infections during pregnancy is mediated by a shift in the population of CD4 + T cells towards the Th2 phenotype compared to Th1, promoting greater humoral responses than cell-type immune responses [14,15,16], along with a decrease in circulating natural killer (NK) cells [17]. In addition, a decrease in circulating plasmacytoid dendritic cells, essential for the production of type 1 interferon against viruses, is observed during pregnancy [18,19]. It should not be forgotten that the increase in circulating progesterone levels, thanks to its immunomodulatory properties, promotes the pulmonary repair of the damage induced by the influenza virus by enhancing recovery after viral lung infections [20,21]. Although the effect of maternal SARS-CoV-2 infection on the newborn is not yet fully understood, recently, Gee et al. have demonstrated similar proportions of B cells, CD4+ and CD8+ T cells and increased percentages of natural killer cells, Vδ2+ γδ T cells and regulatory T cells in infants born to mothers with recent or current infection versus infants born to mothers who are cured or uninfected [22].

Following these considerations, the evaluation of the behavior of the immune system of a women subjected to COVID-19 vaccination during pregnancy acquires even more interest. According to the data presented, the woman was largely positive on the DBS serological test 25 days after the second vaccination dose, but slightly below the medians already described in our previous work [8].

Interestingly, although maternal antibody levels dropped dramatically several months after vaccination, the frequency of memory CD8+ and CD4+ T cells exposing pro-inflammatory cytokines demonstrates that vaccination leads to long-term protection from COVID-19. By monitoring the immune response of a woman receiving the SARS-CoV-2 vaccine during the first trimester of pregnancy, we observed that the mother’s post-vaccination immune response follows the same pattern previously observed in a larger series of people subjected to the same vaccine [8,13].

In agreement with other recent studies, we have observed that the anti-SARS-CoV-2 vaccine transfers antibody immunity to the fetus through the placental barrier, revealing IgG anti-SARS-CoV-2 on neonatal blood samples [11,12,23].

Here, we demonstrate how acquired immunity against SARS-CoV-2, not induced by COVID-19 infection but induced by full vaccination during the first trimester, produced passive fetal immunity, as observed in the first 48 h of life. Interestingly, the value of anti-SARS-CoV-2 IgG antibodies at 48 h of life was even higher than the values measured in the mother 7 months post-vaccination.

A further interesting aspect that the authors would like to underline concerns the use of the DBS samples for the evaluation of IgG anti-SARS-CoV-2 antibody. In fact, serological tests were performed not only on the mother but also on the newborn after 48 h and after 14 days of life. Interestingly, the use of DBS sampling is minimally invasive and able to allow an advantageous alternative to existing analytical procedures such as sampling by venepuncture or nasal swabs. Thanks to the easy collection, it is suitable for self-sampling, for example, in critical situations such as quarantine [24].

Not least, DBS serology tests offer lower sample handling costs, with no need for expensive or time-consuming pre-analytical processes [25]. These features make such tests particularly suitable for close population monitoring. In fact, a similar strategy has been proposed for viral hepatitis screening as well as for human immunodeficiency virus [26,27].

No less important is the use of DBS samples for monitoring about 40 inborn errors of metabolism in expanded neonatal screening programs [28].

This screening test is mandatory in Italy and performed by collecting whole blood from the heel of newborns between 48 and 72 h after birth; the sample is suitable for anti-SARS-CoV-2 IgG testing [29].

## 4. Conclusions

In conclusion, we presented a case of a woman with complete vaccination in the first trimester of pregnancy and a positive response to the vaccine, measured in terms of spike-specific T cell responses.

The serological tests on the DBSs carried out on both mother and newborn revealed the presence of anti-SARS-CoV-2 IgG antibodies up to 7 months after vaccination in the mother and already at 48 h of life in the newborn. The dualism and close collaboration between our COVID-19 center and expanded neonatal screening laboratories allowed the monitoring of the newborn for up to 14 days without the need for an additional and invasive sample collection, exploiting the protocols and time points provided for the screening of metabolic diseases. 

The case we described suggests that it would be of interest and relatively simple to carry out a screening for the detection of anti-SARS-CoV-2 antibodies for all newborns whose mothers have been affected by COVID-19 or have received the vaccine, in order to have a clear picture of the serological characteristic of infants.

## Data Availability

Data are contained within the article.

## Supplementary Materials

The following are available online at https://www.mdpi.com/article/10.3390/ijerph182312789/s1, Table S1: Table reports the standard deviations and CV% of 3 replicates acquired in the same analytical ses-sion (Intra-assay) and 5 replicates acquired in 5 different analysis days (Inter-assay), considering a range of 0.48-35 ratio. Figure S1: Figure shows the values of IgG anti SARS-CoV-2 measured at the 3 time points point: before the BNT162b2 vaccination dose (T0 *n* = 36), 25 days after complete vaccination (*n* = 35) and 7 months after complete vaccination (*n* = 27). The ranges from the 5th to the 95th percentile for each time-point are highlighted on the right. Figure S2: IgG antibodies against SARS-CoV-2 levels measured on two Newborns DBS samples, whose mothers received full administration of the BNT162b2 vaccine in the first trimester of pregnancy, at 2 time-point: at 48 h of life, and 14 days of life. The positivity limit is set to 1.2, as shown by the dashed black line.
