# Supplementary material for: Passive Immunity to SARS-CoV-2 at Birth Induced by Vaccination in the First Trimester of Pregnancy
Source: Int J Environ Res Public Health. 2021 Dec 3;18(23):12789. doi: 10.3390/ijerph182312789 (PMC8657259; doi:10.3390/ijerph182312789)

International Journal of
Environmental Research and Public Health

MDPI

Supplementary Material of Case report

Passive immunity to SARS-CoV-2 at birth induced by vaccination in the first trimester of pregnancy

Ilaria Cicalini [1,2,*], Claudia Rossi[1,3], Luca Natale[1], Maria Concetta Cufaro[1,4], Giulia Catitti[1,5], Simone Vespa[1,5], Domenico De Bellis[1,2], Giulia Iannetti[1], Paola Lanuti[1,5], Ines Bucci[1,5], Liborio Stuppia[1,3], Vincenzo De Laurenzi[1,2] and Damiana Pieragostino[1,2]

[1] Center for Advanced Studies and Technology (CAST), "G. d'Annunzio" University of Chieti-Pescara, 66100 Chieti, Italy; ilaria.cicalini@unich.it (I.C.); claudia.rossi@unich.it (C.R.); lucanatale1989@gmail.com (L.N.); maria.cufaro@unich.it (M.C.C.); catittig@gmail.com (G.C.); sv85@libero.it (S.V.); domenico.debellis@studenti.unich.it (D.D.B); giuliaiannetti686@gmail.com (G.I.), paola.lanuti@unich.it (P.L.); ines.bucci@unich.it (I.B.); stuppia@unich.it (L.S.); delaurenzi@unich.it (V.D.L); damiana.pieragostino@unich.it (D.P)

[2] Department of Innovative Technologies in Medicine and Dentistry, "G. d'Annunzio" University of Chieti-Pescara, 66100 Chieti, Italy;

[3] Department of Psychological, Health and Territory Sciences, School of Medicine and Health Sciences, "G. d'Annunzio" University of Chieti-Pescara, 66100 Chieti, Italy

[4] Department of Pharmacy, "G. d'Annunzio" University of Chieti-Pescara, 66100 Chieti, Italy

[5] Department of Medicine and Aging Science, "G. d'Annunzio" University of Chieti-Pescara, 66100 Chieti, Italy;

* Correspondence: ilaria.cicalini@unich.it; Tel.: +39-0871-541333

Citation: Cicalini I.; Rossi C..; Natale L. et al **Passive immunity to SARS-CoV-2 at birth induced by vaccination in the first trimester of pregnancy**. *Int. J. Environ. Res. Public Health* **2021**, *18*, 12789. https://doi.org/10.3390/ijerph182312789

Academic Editor: Firstname Lastname

Received: date
Accepted: date
Published: 8 December 2021

Publisher's Note: MDPI stays neutral with regard to jurisdictional claims in published maps and institutional affiliations.

Copyright: by the authors. Licensee MDPI, Basel, Switzerland. This article is an open access article distributed under the terms and conditions of the Creative Commons Attribution (CC BY) license (https://creativecommons.org/licenses/by/4.0/).

Abstract: As it is well known, COVID-19 infection is affecting the whole world causing a serious health, social and economic crisis. Viral infection can cause mild or severe illness depending on how effectively the virus is countered by the immune system. In this context, the position of pregnant women remains rather unknown. The case described here reports the immune response in a woman in good health undergoing complete vaccination during the first trimester of pregnancy and in her newborn son. We performed serological assay measuring IgG antibodies to SARS-CoV-2 by a fully automated solid phase DELFIA (time-resolved fluorescence) immunoassay in a few drops of blood collected by finger-prick and spotted on filter paper. The dried blood spot (DBS) sample we used is the same type of sample routinely used in newborn screening program test. Such simple and minimally invasive approach allowed us to monitor both the mother and the newborn soon after birth for anti-SARS-CoV-2 IgG levels. The serological test on DBS carried out on both mother and newborn revealed the presence of anti-SARS-CoV-2 IgG antibodies up to 7 months after vaccination in the mother and already at 48 hours of life in the newborn.

Keywords: SARS-CoV-2, vaccination in pregnancy, newborn SARS-CoV-2 immunity, spike-specific T-cells.

Table S1: Table reports the standard deviations and CV% of 3 replicates acquired in the same analytical session (Intra-assay) and 5 replicates acquired in 5 different analysis days (Inter-assay), considering a range of 0.48-35 ratio.

	**Intra-assay**		**Inter-assay**	
**QC ratio**	SD n=3	CV%	SD N=5	CV%
**0,48**	0,01	**2,08**	0,06	**13,13**
**0,66**	0,02	**3,03**	0,08	**13,98**
**1,03**	0,06	**5,54**	0,13	**14,90**
**1,64**	0,10	**5,82**	0,17	**13,39**
**3,19**	0,13	**4,10**	0,20	**6,98**
**9,02**	0,56	**6,15**	0,69	**8,85**
**20,81**	0,72	**3,47**	2,19	**11,37**
**35,70**	0,09	**0,24**	2,40	**7,22**

Figure S1: Figure shows the values of IgG anti SARS-CoV-2 measured at the 3 time points point: before the BNT162b2 vaccination dose (T0 n = 36), 25 days after complete vaccination (n = 35) and 7 months after complete vaccination (n = 27). The ranges from the 5th to the 95th percentile for each time-point are highlighted on the right.

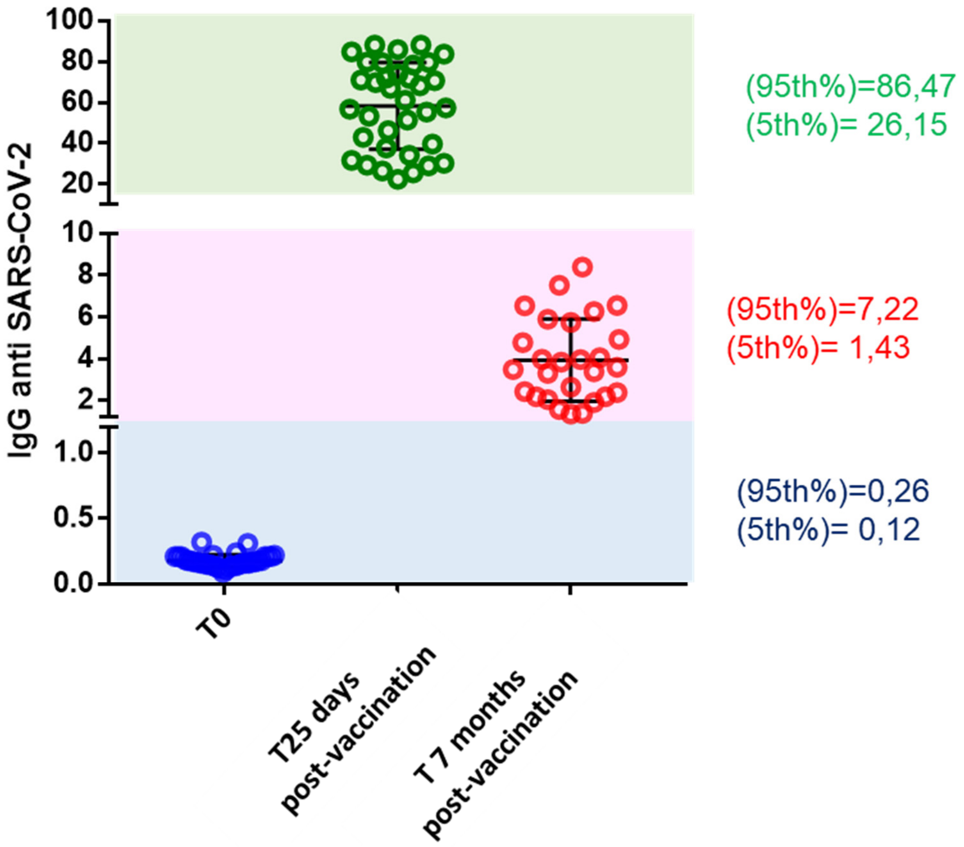

Figure S2: IgG antibodies against SARS-CoV-2 levels measured on two Newborns DBS samples, whose mothers received full administration of the BNT162b2 vaccine in the first trimester of pregnancy, at 2 time-point: at 48h of life, and 14 days of life. The positivity limit is set to 1.2, as shown by the dashed black line.

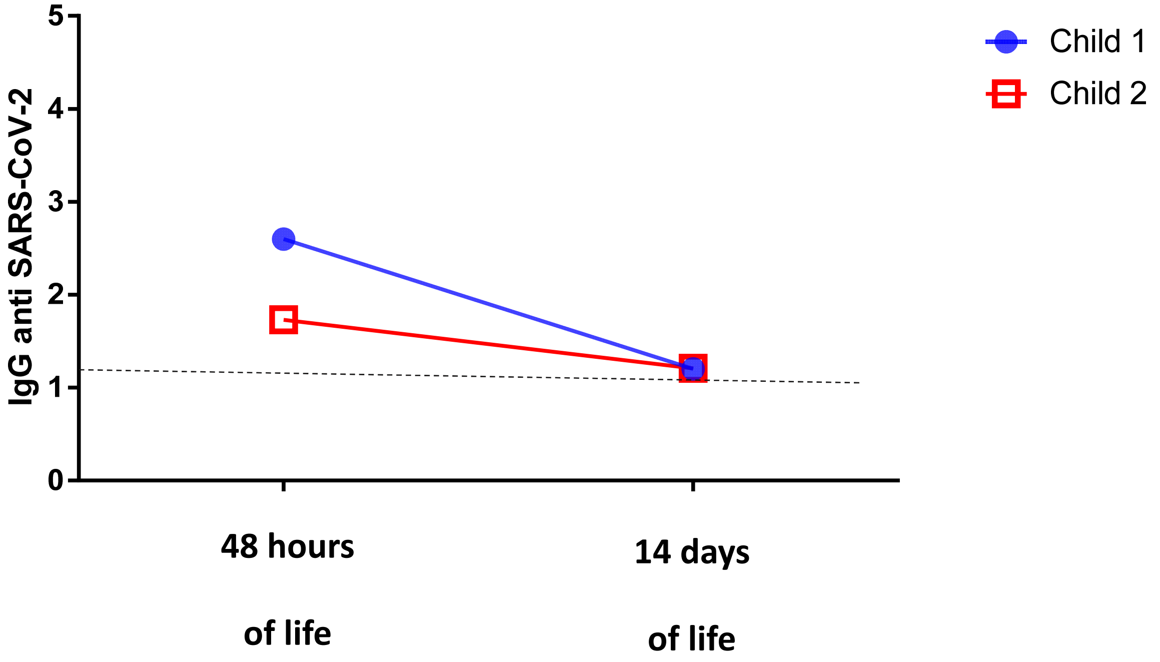

Supplement: Supplementary file 1 [file ijerph-18-12789-s001.zip › ijerph-1489658-supplementary.pdf]
